# Identification and characterization of a bacterial core methionine synthase

**DOI:** 10.1038/s41598-020-58873-z

**Published:** 2020-02-07

**Authors:** Darja Deobald, Rafael Hanna, Shahab Shahryari, Gunhild Layer, Lorenz Adrian

**Affiliations:** 1 Leipzig University, Institute of Biochemistry, Brüderstraße 34, 04103 Leipzig, Germany; 2 Helmholtz Centre for Environmental Research – UFZ, Isotope Biogeochemistry, Permoserstraße 15, 04318 Leipzig, Germany; 3 Freiburg University, Institute of Pharmaceutical Sciences, Stefan-Meier-Straße 19, 79104 Freiburg im Breisgau, Germany; 4 Technische Universität Berlin, Chair of Geobiotechnology, Ackerstraße 76, 13355 Berlin, Germany

**Keywords:** Enzymes, Transferases, Methylases, Molecular evolution

## Abstract

Methionine synthases are essential enzymes for amino acid and methyl group metabolism in all domains of life. Here, we describe a putatively anciently derived type of methionine synthase yet unknown in bacteria, here referred to as core-MetE. The enzyme appears to represent a minimal MetE form and transfers methyl groups from methylcobalamin instead of methyl-tetrahydrofolate to homocysteine. Accordingly, it does not possess the tetrahydrofolate binding domain described for canonical bacterial MetE proteins. In *Dehalococcoides mccartyi* strain CBDB1, an obligate anaerobic, mesophilic, slowly growing organohalide-respiring bacterium, it is encoded by the locus cbdbA481. In line with the observation to not accept methyl groups from methyl-tetrahydrofolate, all known genomes of bacteria of the class *Dehalococcoidia* lack *metF* encoding for methylene-tetrahydrofolate reductase synthesizing methyl-tetrahydrofolate, but all contain a core-*metE* gene. We heterologously expressed core-MetE_CBDB_ in *E. coli* and purified the 38 kDa protein. Core-MetE_CBDB_ exhibited Michaelis-Menten kinetics with respect to methylcob(III)alamin (*K*_M_ ≈ 240 µM) and L-homocysteine (*K*_M_ ≈ 50 µM). Only methylcob(III)alamin was found to be active as methyl donor with a *k*_cat_ ≈ 60 s^−1^. Core-MetE_CBDB_ did not functionally complement *metE*-deficient *E. coli* strain DH5α (*ΔmetE::kan*) suggesting that core-MetE_CBDB_ and the canonical MetE enzyme from *E. coli* have different enzymatic specificities also *in vivo*. Core-MetE appears to be similar to a MetE-ancestor evolved before LUCA (last universal common ancestor) using methylated cobalamins as methyl donor whereas the canonical MetE consists of a tandem repeat and might have evolved by duplication of the core-MetE and diversification of the N-terminal part to a tetrahydrofolate-binding domain.

## Introduction

Methionine plays an essential role as proteinogenic amino acid in all domains of life, as an initiation amino acid in protein translation^1^ and as a precursor in the formation of cysteine, carnitine, taurine and lecithin^2,3^. Moreover, methionine can be converted to *S-*adenosyl-L-methionine (SAM)^4^, which represents an activated methyl group donor for many fundamental cellular processes^5,6^. The final step in methionine *de novo* synthesis, the methylation of homocysteine to methionine, is catalyzed by different types of methionine synthases including cobalamin-dependent (MetH) and cobalamin-independent methionine synthase (MetE). Some bacteria, *e.g. Escherichia coli*, possess genes for both enzymes^7^ and repress the expression of *metE* in the presence of vitamin B_12_^8^. Homocysteine methylation in mammals is catalyzed by mammalian methionine synthases (mMS) similar to bacterial MetH^9^, betaine-L-homocysteine-*S*-methyltransferase (BHMT) or *S-*methyl-L-methionine-L-homocysteine-*S*-methyltransferase (also known as BHMT-2)^10^. Fungi and plants encode exclusively MetE^11^ or BHMT-2^12^. All known methionine synthase types contain a zinc ion in the active site that is essential for homocysteine binding and methyl group transfer^13^.

MetH (EC 2.1.1.13) catalyzes the methyl transfer from 5-methyl-tetrahydrofolate-monoglutamate (5-methyl-THF-Glu) to homocysteine. MetH from *E. coli* is a large monomeric protein of 1,227 amino acids (136 kDa) and is composed of four functional domains^14^. In the catalytic cycle the methyl group of methylcob(III)alamin is transferred to homocysteine forming cob(I)alamin and methionine. Subsequently, cob(I)alamin is remethylated using 5-methyl-THF-Glu as the methyl group donor regenerating methylcob(III)alamin. For reactivation of cob(II)alamin to methylcob(III)alamin, which is generated in a side-reaction approximately once in 2,000 turnovers^15^, SAM is required^16^.

**Figure 1:**
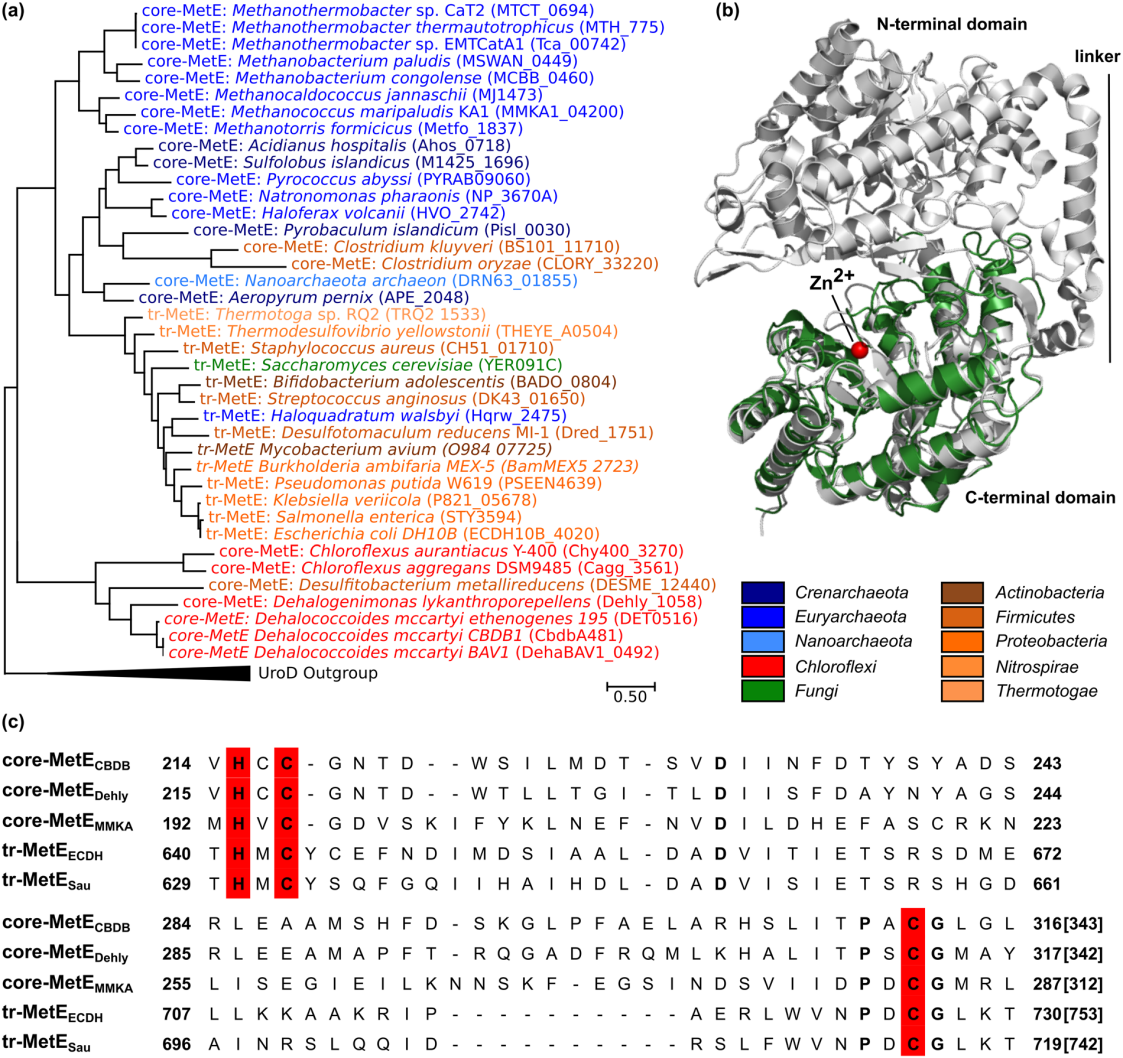
Bioinformatic analysis of the core-MetE_CBDB_ from *Dehalococcoides mccartyi* strain CBDB1. **(a)** Maximum-Likelihood phylogenetic tree of MetE representatives was generated with MEGA7^67^. Multiple amino acid sequence alignments of full length with deletion of gaps (MUSCLE algorithm) were used to generate the tree. The analysis involved 39 amino acid sequences including the C-terminus of tandem-repeat methionine synthases (tr-MetE) from bacteria (brown colors) and yeast (green) as well as core-MetEs from archaea (blue colors), *Chloroflexi* (red) and *Clostridiales* (brown). The gene loci are in brackets. **(b)** The crystal structure of core-MetE_CBDB_ was calculated with the I-TASSER server^65^. Overlay of the crystal structure of tr-MetE from *Neurospora crassa* (PDB No. 4ZTX, grey) with the structural model obtained for core-MetE_CBDB_ from *D. mccartyi* strain CBDB1 (green) was obtained with PyMOL^66^. Core-MetE_CBDB_ matches the C-terminal part of tr-MetE_Ncra_ (C-score = −0.27) but lacks the N-terminal part and the linker region. **(c)** Amino acid sequence alignment of selected MetE proteins. The Zn^2+^-binding site HXCX_n_C (red) is conserved in annotated tr-MetEs and also in core-MetE homologs. Core-MetE_CBDB_: core-MetE from *D. mccartyi* strain CBDB1, coreMetE_Dehly_: core-MetE from *Dehalogenimonas lykanthroporepellens*, core-MetE_MMKA_: core-MetE from *Methanococcus maripaludis*, tr-MetE_ECDH_: tandem-repeat MetE from *Escherichia coli* DH10B, tr-MetE_Sau_: tandem-repeat MetE from *Staphylococcus aureus*.

Canonical MetE proteins (EC 2.1.1.14) are described as a family of zinc-containing metalloenzymes sharing no sequence similarity with MetH^1,13^. They catalyze the methylation of homocysteine using 5-methyl-THF-Glu_n_ (n ≥ 3) as methyl donor without the involvement of cobalamin. MetE in *E. coli* is a protein of 753 amino acid residues (85 kDa) that is composed of two homologous parts connected by a linker region (Fig. 1b), suggesting that the domains have evolved by gene duplication of a sequence encoding a smaller protein of approximately 340 amino acid residues^17–19^. We here refer to the canonical *E. coli*-type MetE as ‘tandem-repeat MetE’ (tr-MetE). The active site of tr-MetE is located within the C-terminal part, where the zinc ion is coordinated by one histidine, two cysteine and one glutamate residue. The binding site for methyl-THF is in the cleft between the two domains of tr-MetE^19^.

*Dehalococcoides mccartyi* strain CBDB1 is an obligately anaerobic, mesophilic bacterium belonging to the phylum *Chloroflexi*, class *Dehalococcoidia*^20,21^. *Dehalococcoides* species are well known for their ability to use a wide range of persistent and toxic halogenated organic compounds as terminal electron acceptor in anaerobic respiration (“organohalide respiration”) with hydrogen as electron donor^22^. Strain CBDB1 encodes one of the largest numbers of B_12_-dependent proteins in known prokaryotes^23^. The most prominent representatives of B_12_-dependent proteins in strain CBDB1 are reductive dehalogenases that are responsible for the reduction of halogenated pollutants as a terminal electron acceptor^24^. Vitamin B_12_ in the medium is essential for the growth of *Dehalococcoides* strains^25^ because *Dehalococcoides* do not contain many of the genes for *de novo* biosynthesis of cobalamin^26,27^. However, *Dehalococcoides* strains encode corrinoid-specific ABC-transporter and enzymes for the late B_12_ biosynthesis pathway enabling them to incorporate corrinoid precursors from the environment and to modify them to cobalamin^28,29^. Although none of the known *D. mccartyi* genomes contains full gene homologs of *metE*, *metH, bhmt* or *bhmt-2*^26^, *D. mccartyi* strains synthesize methionine *de novo*^24,30,31^. Zhuang *et al*. demonstrated that acetyl-CoA donates the C_2_-methyl group for an unconventional methionine biosynthesis pathway independent from methylene-tetrahydrofolate reductase (MTHFR). All *D. mccartyi* species sequenced so far, lack *metF* encoding for MTHFR that reduces 5,10-methylene-THF-Glu to 5-methyl-THF-Glu. Furthermore, *D. mccartyi* strain 195 was not able to incorporate 5-methyl-THF from the environment^32^.

Here, we found that locus cbdbA481 (NCBI accession number CAI82680) of *D. mccartyi* strain CBDB1 encodes a 343 amino acid protein that is homologous to the C-terminus of canonical tr-MetE and similar to methylcobalamin:homocysteine methyltransferase (core-MetE_MTH_) of the methanogenic archaeum *Methanobacterium thermoautotrophicum*^33^. In our study we provide biochemical and genetic evidence that the locus cbdbA481 encodes a novel type of bacterial methionine synthase that appears to be an anciently derived MetE-related methionine synthase obtaining its methyl group from an external corrinoid rather than from folate. Together with archaeal methylcobalamin:homocysteine methyltransferases and bacterial homologs the gene product of locus cbdbA481 forms a new group of basal methionine synthases, referred to as core-MetE in the following.

## Results

### Bioinformatic analysis of locus cbdbA481 in the genome of *D. mccartyi* strain CBDB1

*D. mccartyi* strains are able to synthesize methionine *de novo*, although *D. mccartyi* genomes do not contain gene homologs of *metE, metH, bhmt* or *bhmt*-2^26^. In the KEGG (Kyoto Encyclopedia of Genes and Genomes) database several enzymes of the *Dehalococcoides* methionine metabolism are annotated^34–36^. The loci cbdbA476 and cbdbA477 in the genome of strain CBDB1 are annotated as SAM synthetase (EC 2.5.1.6) and *S*-adenosyl-L-homocysteinase (EC 3.3.1.1), respectively. Since methionine biosynthesis and SAM metabolism are biochemically closely linked, we started our search for a methionine synthase gene in the genome of strain CBDB1 by inspecting the direct neighborhood of the loci cbdbA476 and cbdbA477. Locus cbdbA481 located in the same operon encodes a 343 amino acid polypeptide (here referred to as core-MetE_CBDB_) with a calculated molecular mass of 38 kDa that has sequence similarity with the C-terminal half of canonical tandem-repeat MetE (tr-MetE) proteins. Accordingly, the calculated mass of core-MetE_CBDB_ is only about half the size of tr-MetE proteins (*e.g*. tr-MetE_Eco_ of *E. coli*). Phylogenetic analysis of core-MetE_CBDB_ together with 38 other protein sequences including annotated tr-MetE representatives, archaeal methylcobalamin:homocysteine methyltransferase homologs (core-MetE_Archaea_) and *Chloroflexi* sequences with high sequence similarity to core-MetE_CBDB_ indicates that core-MetE_CBDB_ is the prototype of a new bacterial cluster of short MetE sequences (Fig. 1a). This new cluster is phylogenetically well separated from the C-termini of tr-MetE proteins and core-MetE_Archaea_ (Fig. 1a, blue colors)^33^. Core-MetE proteins are widely distributed in microorganisms with strongly conserved ancient traits (archaea, *Clostridiales, Dehalococcoidia* and *Chloroflexia* classes). Within the archaea, only *Haloquadratum* spp. encode the tr-MetE, whereas many archaea encode a core-MetE homolog (Fig. 1a, blue colors). Bacterial and archaeal core-MetE form a paraphyletic group (excluding the tr-MetE sequences), probably evolved before LUCA (last universal common ancestor) and branched into two groups. Tr-MetEs appear to have evolved from archaeal core-MetE (*e.g*. core-MetE in *Clostridium kluyveri* and *C. oryzae*) (Fig. 1a).

The computational structural model of core-MetE_CBDB_ (Fig. 1b, green) resembles the C-terminal domain of annotated tr-MetE proteins, bearing the highest similarity to methionine synthase from *Neurospora crassa* (C-score = −0.27, identity = 21% and RMSD = 3.52) (PDB No. 4ZTX)^37^. Compared to tr-MetE proteins, core-MetE_CBDB_ lacks the N-terminal domain described to be responsible for 5-methyl-THF binding and the linker region between the C- and N-terminal parts (Fig. 1b, grey). An amino acid alignment of core-MetE_CBDB_ with core-MetE_Dehly_ from *Dehalogenimonas lykanthroporepellens*, core-MetE_MMKA_ from *Methanococcus maripaludis* KA1 and the C-terminal halves of the tr-MetE_ECDH_ from *E. coli* DH10B and tr-MetE_Sau_ from *Staphylococcus aureus* shows that the zinc binding motif HXCX_n_C, essential for L-homocysteine binding and activation^13^, is conserved in core-MetE_CBDB_ (Fig. 1c). The computational model indicates that in strain CBDB1, zinc is coordinated in a tetrahedral fashion by His215, Cys217 and Cys312 (Fig. 1c) which are conserved among all MetEs and by Asp236. In contrast, zinc of tr-MetE from *N. crassa* is bound by histidine, two cysteine and one glutamate residue.

### Heterologous production and purification of core-MetE_CBDB_

To study the function of core-MetE_CBDB_ in detail, the recombinant protein was heterologously produced in *E. coli* and purified. First, production and purification attempts were conducted for a C-terminally Streptavidin-tagged core-MetE_CBDB_ using affinity chromatography for purification. However, native polyacrylamide gel electrophoresis (PAGE) indicated misfolding of the protein (Supplementary Figure 1a,b). Therefore, core-MetE_CBDB_ was produced without a tag in *E. coli* and purified using anion exchange chromatography. The purified protein exhibited a molecular mass of approximately 38 kDa, as determined by SDS-PAGE (Supplementary Figure 1c). The identity of core-MetE_CBDB_ was verified by liquid chromatography-tandem mass spectrometry (LC-MS/MS) (47 validated unique peptides, 87% coverage). Purified core-MetE_CBDB_ was in its monomeric form as indicated by native PAGE (Supplementary Figure 1d) and analytical size exclusion chromatography (data not shown).

### Coordination environment of methylcob(III)alamin bound to core-MetE_CBDB_

**Figure 2:**
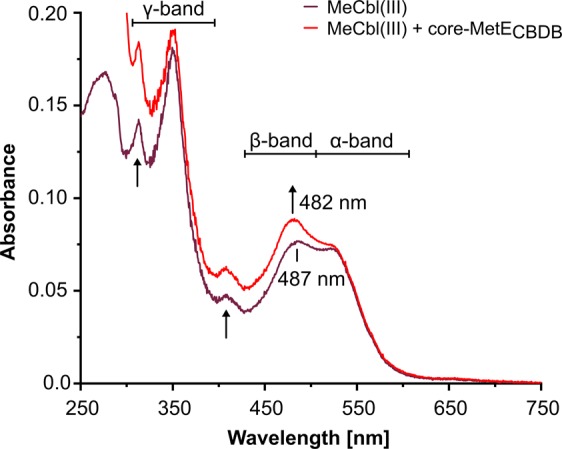
Coordination mode of methylcob(III)alamin (MeCbl(III)) bound to core-MetE_CBDB_ from *Dehalococcoides mccartyi* strain CBDB1. Free MeCbl(III) in the “base-on” mode is characterized by broad α/β-absorbance bands and characteristic maxima at ~ 487 and 524 nm (violet line). The UV/Vis spectrum of MeCbl(III) in the presence of core-MetE_CBDB_ at 1:1 stoichiometry was slightly changed. The absorbance intensities of β- and γ-bands increased and the maximum of the β-band shifted (Δλ = −5 nm) (red line), indicating the “base-off/His-on” binding mode of MeCbl(III).

The binding of methylcob(III)alamin to core-MetE_CBDB_ was analyzed spectrophotometrically after mixing methylcob(III)alamin and core-MetE_CBDB_ in a 1:1 stoichiometry at a concentration of 10 µM each. Free methylcob(III)alamin exhibited α/β- and γ-absorption bands characteristic for the “base-on” mode (Fig. 2, violet line)^38^. On binding to core-MetE_CBDB_, the UV/Vis spectrum of methylcob(III)alamin slightly changed: the absorption intensities of β- and γ-bands increased and the absorption maximum of the β-band blue-shifted slightly (Δλ = −5 nm) (Fig. 2, red line).

### Core-MetE_CBDB_ catalyzes methionine formation with methylcob(III)alamin as methyl donor

**Figure 3:**
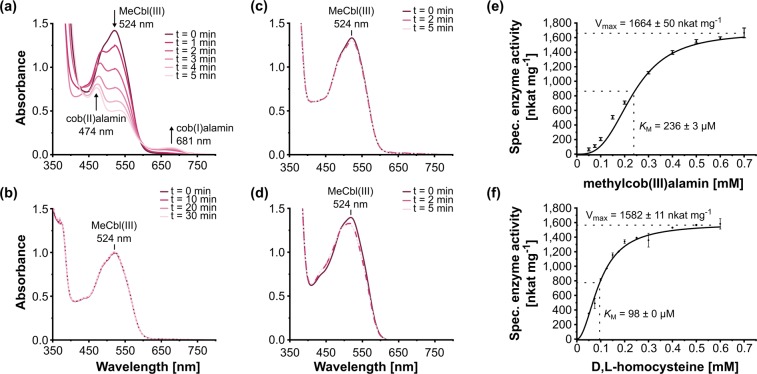
Demethylation of methylcob(III)alamin (MeCbl(III)) catalyzed by purified core-MetE_CBDB_ from *D. mccartyi* strain CBDB1 in the presence of D,L-homocysteine. (**a**) In the presence of 0.1 µM core-MetE_CBDB_, 0.5 mM methylcob(III)alamin and 2 mM D,L-homocysteine, continuous changes in the UV/Vis spectrum were observed indicating the consumption of methylcob(III)alamin (524 nm) and the formation of cob(I)alamin (681 nm) and cob(II)alamin (474 nm) as highlighted by arrows. (**b**) In the absence of core-MetE_CBDB_, the UV/Vis spectrum remained unchanged over an incubation time of 30 min. (**c**) In the absence of D,L-homocysteine, the UV/Vis spectrum remained unchanged. (**d**) In the presence of 0.5 mg mL^−1^ *E. coli* crude extract, instead of core-MetE_CBDB_ as catalyst, MeCbl(III) was not transformed. (**e**) Dependence of core-MetE_CBDB_ methyltransferase activity on methylcob(III)alamin concentration. (**f**) Dependence of core-MetE_CBDB_ methyltransferase activity on D,L-homocysteine concentration. The activities in panels (**e**,**f**) were determined by following the change of the absorption at 681 nm. *V*_max_ and *K*_M_ were calculated according to a Hill-Fit plot with R^2^ = 0.998 for panel (**e**) and R^2^ = 0.999 for panel (**f**), respectively.

The enzymatic activity of purified core-MetE_CBDB_ was tested using methylcob(III)alamin as methyl donor and homocysteine as methyl acceptor. In the presence of core-MetE_CBDB_, the UV/Vis absorption spectrum of methylcob(III)alamin, exhibiting a characteristic maximum at 524 nm, successively changed over time due to the consumption of methylcob(III)alamin and formation of cob(I)alamin and cob(II)alamin, as indicated by the emergence of absorption features at 681 nm and 474 nm, respectively (Fig. 3a). In the absence of core-MetE_CBDB_ or homocysteine, the UV/Vis spectrum of methylcob(III)alamin remained unchanged (Fig. 3b,c). In order to exclude any methyltransferase activity due to impurities of the protein preparation, *E. coli* cell-free extract was also tested and did not show any activity (Fig. 3d). Finally, in addition to the photometric measurements, the formation of methionine ([M + H]+  = 150.0583 m/z) during the enzymatic reaction was verified *via* liquid chromatography-mass spectrometry (LC-MS) (Supplementary Figure 2b). In the following, core-MetE_CBDB_ enzyme activity was monitored by measuring the increase of absorption at 681 nm (Fig. 3e,f) or the decrease of absorption at 524 nm (Supplementary Figure 2a). Kinetic parameters for core-MetE_CBDB_ were determined using an enzyme concentration of 0.1 µM. At a constant D,L-homocysteine concentration of 2 mM and varying methylcob(III)alamin concentrations, methionine was formed with a *V*_max_ = 1664 ± 50 nkat mg^−1^ and a *K*_M_ = 236 ± 3 µM for methylcob(III)alamin (Fig. 3e). When different D,L-homocysteine concentrations were used at a fixed methylcob(III)alamin concentration of 0.5 mM, a *V*_max_ = 1582 ± 11 nkat mg^−1^ and a *K*_M_ = 98 ± 0 µM for D,L-homocysteine were estimated (Fig. 3f). Since methionine synthase is specific for L-homocysteine, the apparent *K*_M_ for L-homocysteine might be half of that for D,L-homocysteine^33^. The maximum turnover number (*k*_cat_) was calculated to be about 60 s^−1^. The substrate specificity of core-MetE_CBDB_ was investigated by replacing homocysteine with 2 mM cysteine, 2 mM glutathione or 2 mM dithiothreitol. Core-MetE_CBDB_ did not show any activity towards these thiol analogs (data not shown).

**Figure 4:**
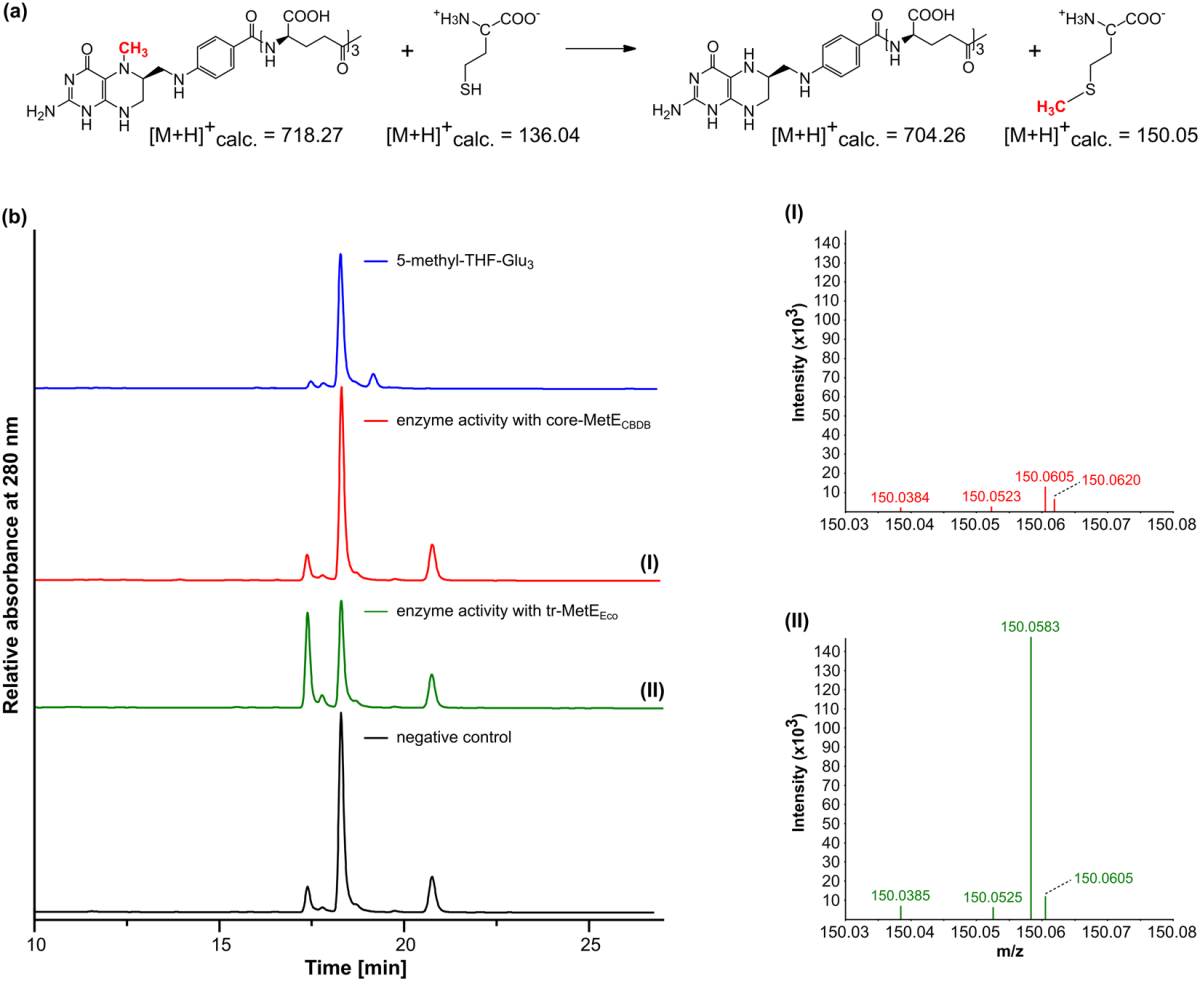
Core-MetE_CBDB_ from *D. mccartyi* strain CBDB1 does not catalyze the formation of L-methionine with 5-methyl-THF-Glu_3_ as the methyl donor. (**a**) Reaction described for tr-MetE from *E. coli* (tr-MetE_Eco_) catalyzing the methylation of L-homocysteine with 5-methyl-THF-Glu_3_ to form L-methionine and THF-Glu_3_^80^. (**b**) Representative HPLC chromatograms of a chemical standard of 5-methyl-THF-Glu_3_ (blue), of reaction products after an enzyme activity assay containing 5-methyl-THF-Glu_3_, D,L-homocysteine and either core-MetE_CBDB_ (red) or tr-MetE_Eco_ (green) or no enzyme (black). The peak at RT = 21 min represents dithiothreitol added to all reactions. In the presence of tr-MetE_Eco_, 5-methyl-THF-Glu_3_ (RT = 18 min) reacts to form THF-Glu_3_ (RT = 17.4 min). Slow demethylation of 5-methyl-THF-Glu_3_ to THF-Glu_3_ did occur in the negative control and also in the presence of core-MetE_CBDB_. To evaluate if this demethylation was linked to L-methionine formation, the products of the activity assays were analyzed by mass spectrometry. I) No L-methionine was formed in the presence of core-MetE_CBDB_; II) The product of tr-MetE_Eco_ was identified as L-methionine ([M + H]+  = 150.0583 m/z).

Additionally, 5-methyl-THF-Glu_3_ was tested as a methyl group donor for core-MetE_CBDB_ instead of methylcobalamin (Fig. 4b). In the negative control and also in the presence of core-MetE_CBDB_, slow demethylation of 5 methyl-THF-Glu_3_ occurred abiotically^39–41^. The demethylation of 5-methyl-THF-Glu_3_ in the negative control and in the presence of core-MetE_CBDB_ was not linked to L methionine formation (Fig. 4b(I)), while in the presence of tr-MetE_Eco_, methionine was formed exhibiting a signal at [M + H]^+^  = 150.0583 m/z (Fig. 4b(II)).

### pH-optimum and thermal stability of core-MetE_CBDB_

Methionine synthase activity of core-MetE_CBDB_ was observed between pH 5.0 and 9.0, with an optimum between pH 6 and 6.5 (Table 1). The thermal stability of purified tr-MetE_Eco_ and core-MetE_CBDB_ were assessed by recording protein melting curves using nano differential scanning fluorimetry (nanoDSF). For tr-MetE_Eco_, a melting temperature T_m_ = 55.8 ± 0.2 °C was determined. In contrast, the T_m_ of core-MetE_CBDB_ was at 68.8 ± 0.0 °C (Supplementary Figure 3).

**Table 1:** Demethylation of methylcob(III)alamin (MeCbl(III)) catalyzed by core-MetE_CBDB_ from *D. mccartyi* strain CBDB1 in the presence of D,L-homocysteine at different pH values.

pH	MeCbl(III) consumption [µM min^−1^]	relative activity [%]
5.0	20.53 ± 4.46	82.0
5.5	19.12 ± 3.23	76.4
6.0	25.05 ± 0.37	100
6.5	23.87 ± 1.41	95.3
7.0	18.96 ± 0.07	75.6
7.5	21.61 ± 0.41	86.3
8.0	18.08 ± 1.02	72.3
8.5	17.70 ± 0.88	70.6
9.0	14.23 ± 0.61	56.9

### Core-MetE_CBDB_ does not complement tr-*metE*-deficient *E. coli in vivo*

The examination of enzymatic activities of core-MetE_CBDB_ *in vitro* has limitations. However, we were not able to conduct *in vivo* mutagenesis studies with strain CBDB1 because *Dehalococcoide*s species are not yet genetically accessible. In order to obtain insights into the physiological role of the cbdbA481 gene product *in vivo*, we tested whether a tr-*metE*-deficient *E. coli* strain could be complemented by core-MetE_CBDB_. Therefore, we generated a tr-*metE*-deficient knockout strain of *E. coli* DH5α (*ΔmetE::kan*) that still contained the *metH* gene for the cobalamin-dependent MetH. This strain was not able to grow in medium without added cyanocobalamin (Supplementary Figure 4, red solid line), but grew when cyanocobalamin was supplemented (Supplementary Figure 4, red dotted line). Next, the growth behavior of the mutant strain carrying different complementation plasmids was investigated. Either the original tr-*metE*_Eco_ gene or the core-MetE_CBDB_ nucleotide sequence, both under the control of an arabinose promotor, were provided. Growth experiments with these complementation strains showed that neither of the two strains grew without inducing gene expression by arabinose. After induction with arabinose, tr-*metE*_Eco_ was able to complement the *ΔmetE* strain as expected (Supplementary Figure 4, blue dotted line), while core-MetE_CBDB_ was not (Supplementary Figure 4, green dotted line). These results suggested that core-MetE_CBDB_ does not have the same physiological function as the canonical tr-MetE_Eco_.

## Discussion

Methionine and SAM have been suggested to belong to the most ancient molecules on earth and might have emerged within or even before the “RNA world”^42–44^. Although methionine appears to have a continued central metabolic role for more than three billion years, different routes for its biosynthesis have evolved. The biochemically conserved methionine pathway appears to be the product of an evolutionary patchwork involving diverse methionine synthases^5^. In our study, we identified a novel bacterial MetE-like methionine synthase in *D. mccartyi* strain CBDB1 that uses methylcobalamin as methyl donor instead of methylated tetrahydrofolate. Our results suggest that this enzyme is the basal form of canonical tandem-repeat MetE (tr-MetE) proteins with roughly half its size and without the domain duplication of canonical MetE proteins evolved to enable tetrahydrofolate binding on the N-terminal domain^19^. Homologs of this short methionine synthase are encoded in the genomes of several deeply-rooting obligate anaerobic microorganisms from both prokaryotic domains, including all *Dehalococcoidia* and many *Clostridia* (*e.g. Desulfitobacterium metallireducens, C. kluyveri, C. oryzae*) as well as almost all archaea sequenced so far (Fig. 1a). We refer to this short monomeric MetE form as “core-MetE”, because several lines of evidence hint at its basal descendence including the lack of duplication, the exclusive presence in deeply rooting phylogenetic taxa, and the dependence on corrinoids, which are thought to be ancient cofactors^45,46^, as they participate in fundamental processes such as ribonucleotide reduction^47^, the Wood-Ljungdahl-pathway^48^ and methane formation^49^.

**Figure 5:**
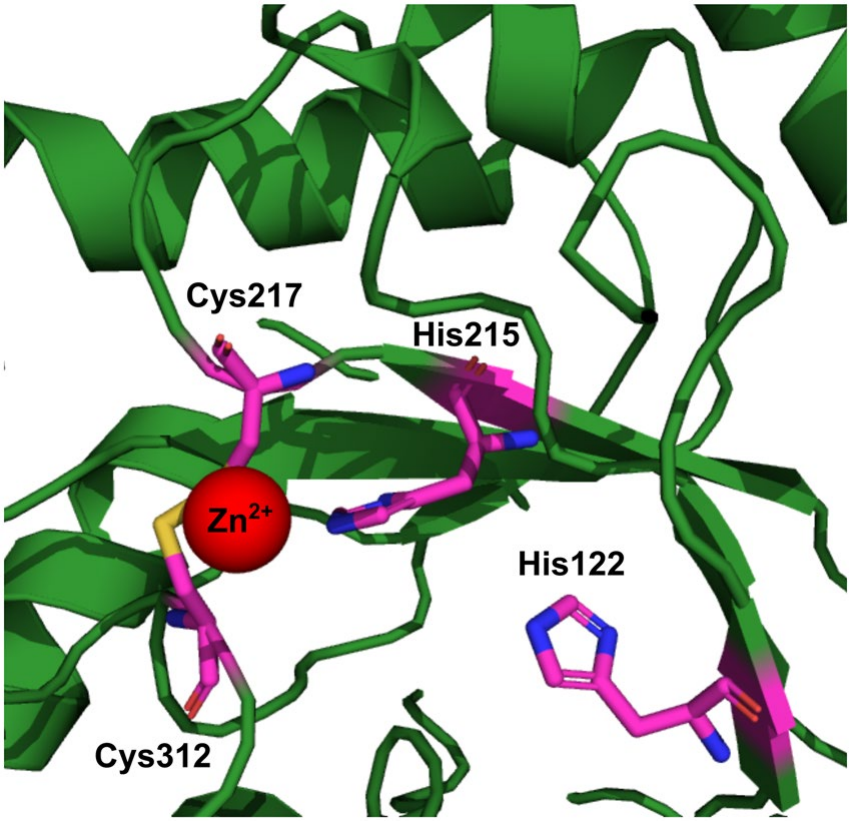
Computational model of the active site of core-MetE_CBDB_ from *Dehalococcoides mccartyi* strain CBDB1. His122 is tuned towards the active site of the protein where a zinc atom is coordinated by His215, Cys217 and Cys312. His122 might replace the dimethylbenzimidazole moiety of cobalamin in the “bae-off/His-on” mode. The structure was calculated with the I-TASSER server^65^ and visualized with PyMOL^66^.

Compared with tr-MetE_Eco_, core-MetE_CBDB_ is more stable towards pH changes^18^ and thermal denaturation. The turnover number *k*_cat_ ≈ 60 s^−1^ of core-MetE_CBDB_ is very high in comparison to other methionine synthases such as tr-MetE_Eco_ with 0.4 s^−1^ ^50^ or *E. coli* MetH with 26 s^−1^ ^51^. The activity of MetH is based on domain movements, which could contribute to the lower catalytic rate in comparison to a small monomeric core-MetE. The relatively slow conversion rate of tr-MetE proteins can be due to the poor methylation power of 5-methyl-THF-Glu_n_ (Fig. 4a) and the weak nucleophilicity of homocysteine at physiological pH^52^. In tr-MetE and MetH, 5-methyl-THF must be activated for the nucleophilic attack by protonation at N^5^ ^15^. In both MetE and MetH, the nucleophilicity of homocysteine is enhanced by coordination with Zn^2+^ that serves as Lewis acid^13^. While in the “base-on” form the dimethylbenzimidazole (Dmbz) base of methylcob(III)alamin is coordinated to the cobalt center of the corrin ring, in the “base-off” mode Dmbz is dissociated from the cobalt. Stabilization of the transition state of methylcob(III)alamin in the “base-off” or “base-off/His-on” binding mode enable nucleophilic attack of homocysteine by weakening the Co-C bond and by reducing the thermodynamic barrier^53–55^. Thus, only binding modes “base-off” or “base-off/His-on” enable methyl transfer from methylcob(III)alamin. However, the “base-off” mode of methylcob(III)alamin which is characterized by strong spectral changes namely, a significant blue shift in the UV/Vis spectrum and reduced intensity of the γ-band^38,56,57^, was not observed in our study (Fig. 2). It is difficult to precisely distinguish between the “base-on” and “base-off/His-on” form because, the UV/Vis spectra of them are very similar^56^. The formation of methionine and cob(I)alamin (Fig. 3a) can only take place if methylcob(III)alamin and homocysteine are bound to core-MetE_CBDB_ in a stable and catalytically favorable configuration. Due to the minor spectral changes, we propose that methylcob(III)alamin is utilized by core-MetE_CBDB_ in the “base-off/His-on” binding mode. The “base-off/His-on” binding mode is found in many B_12_-dependent proteins with a consensus motif Dx**H**xxG, where His represents the lower axial ligand replacing the Dmbz moiety^58^. In our computational model of core-MetE_CBDB_, His122 points towards the active site of the protein and probably belongs to a truncated B_12_-binding motif with the sequence **H**xxG, conserved among all *Dehalococcoidia* (Fig. 5).

The determined *K*_M_-value of core-MetE_CBDB_ for methylcob(III)alamin of ~ 240 µM is likely much higher than the intracellular concentration of free methylcobalamin^29,59^. Therefore, the physiological methyl donor might not be soluble methyl(III)cobalamin. We hypothesize that the physiological methyl donor is a corrinoid protein that directly interacts with core-MetE_CBDB_. Needless to say, that inference of physiological characteristics from the determination of enzyme activity *in vitro* is limited. Examining the role of core-MetE *in vivo* could shed more light on the essentiality and functionality of the enzyme. However, genetic modification of *Dehalococcoides* strains is not possible yet.

**Figure 6:**
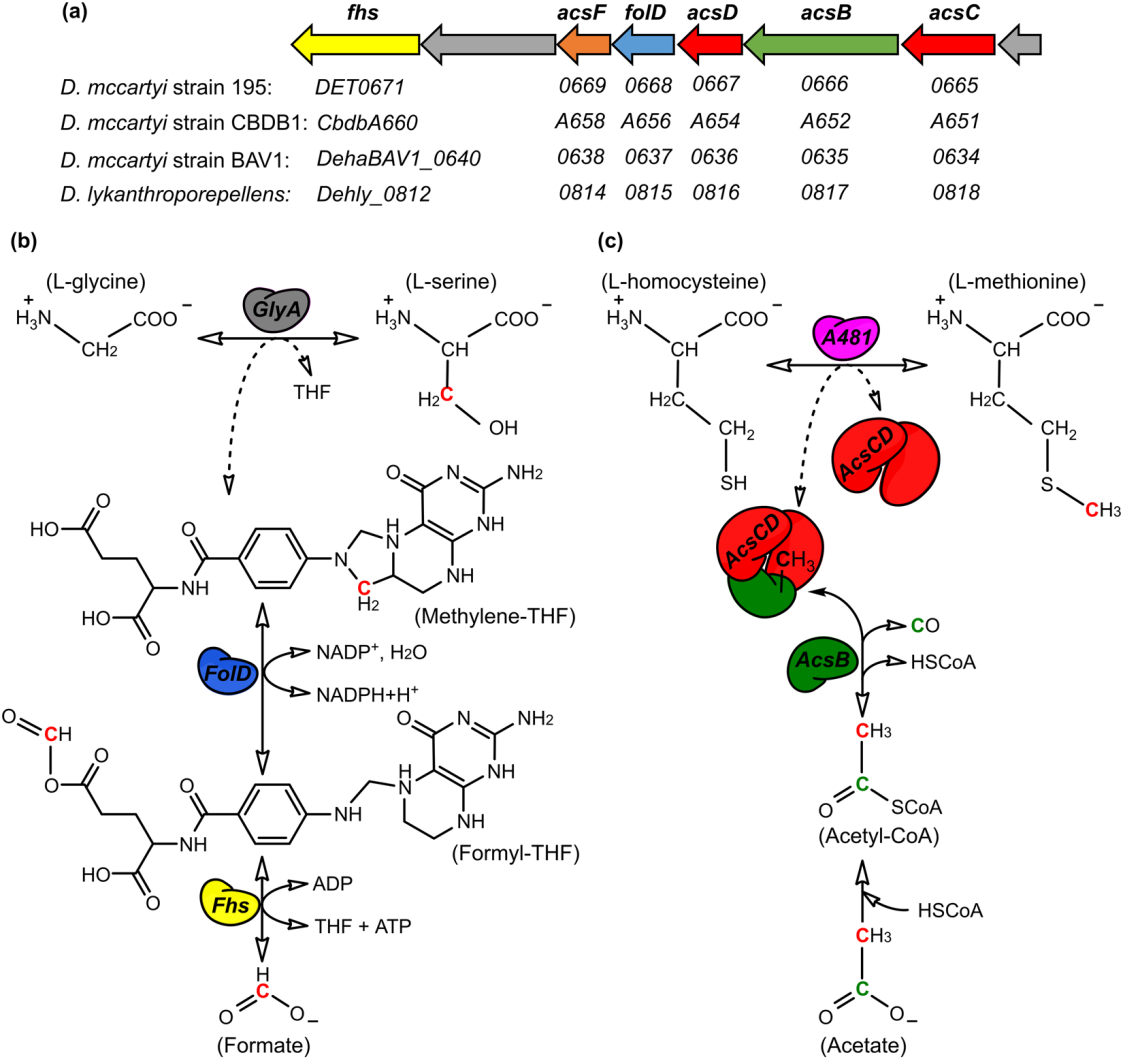
Pathways of L-glycine and L-homocysteine methylation in *Dehalococcoides* species and hypothesized involvement of THF and corrinoid proteins. (**a**) Genes annotated to be involved in the incomplete Wood-Ljungdahl pathway encoded in *D. mccartyi* strain 195 and the respective homologous genes in other *Dehalococcoidia*. (**b**) L-serine formation *via* glycine hydroxymethyltransferase (GlyA, grey) as proposed by Zhuang *et al*. is shown^32^. The methyl group is derived most probably from formate with the aid of formyl-tetrahydrofolate synthase (Fhs, yellow) and methylene-tetrahydrofolate dehydrogenase/cyclohydrolase (FolD, blue). (**c**) Methylation of L-homocysteine is conducted by core-MetE_CBDB_ encoded by the locus cbdbA481 (purple) in *D. mccartyi* strain CBDB1. The original source of the methyl group is acetate, which is activated to acetyl-CoA and then cleaved by acetyl-CoA decarbonylase (AcsB, green) into HSCoA, carbon monoxide (CO) and a methyl group. The standard activity of AcsB is to transfer the methyl group to a corrinoid iron-sulfur protein complex (CoFeSP) AcsCD (red). We speculate that the methyl group is directly transferred from the CoFeSP to the core-MetE_CBDB_ (A481) for L-homocysteine methylation (dashed arrow) but this transfer could also be indirect *via* a yet unidentified participant.

The methyl group transferred by *Dehalococcoides* methionine synthase origins from exogenously supplied acetate, as has been shown by Zhuang *et al*.^32^. Acetate is activated in *Dehalococcoides* by acetyl-CoA synthetase (ACS) to acetyl-CoA^31^. Acetyl-CoA is then cleaved to free coenzyme A, carbon monoxide (which leaves the cell) and a methyl group originating from the C_2_-atom of acetate. This reaction is catalyzed by acetyl-CoA decarbonylase/synthase (AcsB), an enzyme known mostly for its activity in the opposite direction for carbon fixation *via* the Wood-Ljungdahl pathway^60^. In *D. mccartyi* strain CBDB1, AcsB represents the acetyl-CoA decarbonylase and AcsCD a dimeric corrinoid iron-sulfur protein (CoFeSP) to which the methyl group from acetyl-CoA is transferred^61^. Zhuang *et al*. hypothesized that the methyl group is then transferred from AcsCD to tetrahydrofolate and from there to homocysteine, but *Dehalococcoides* neither encode the methyltransferases *acsE*, responsible for the methyl transfer from CoFeSP to tetrahydrofolate nor the classical *metE*/*metH*, responsible for methyl transfer from methyl-tetrahydrofolate to homocysteine (Fig. 6a,b)^32^. Our results can now explain these two gaps by hypothesizing that the methyl group from AcsCD/CoFeSP is directly transferred to homocysteine by core-MetE_CBDB_ instead of taking the diversion *via* tetrahydrofolate (Fig. 6c). This hypothesis would also explain the absence of carbon monoxide dehydrogenase in *Dehalococcoides* which would be needed if the Wood-Ljungdahl pathway was employed for CO_2_ fixation. With the direct transfer of methyl groups from CoFeSP to homocysteine, the cells would be independent from methyl-tetrahydrofolate and indeed *metF* encoding methylene-tetrahydrofolate reductase (MTHFR) is missing in all *Dehalococcoides* genomes^27^. This might be an unusual pathway in extant microbiology but in our view could represent a very early evolutionary stage in which methyl metabolism could have been independent from folates. This view is supported by the fact that methionine, SAM, corrinoids and coenzyme A are conserved between archaea and bacteria but tetrahydrofolate/tetrahydromethanopterin are not^45,46^.

Enzymes similar to the core-MetE identified in *Dehalococcoides* were also found in the majority of archaea (Fig. 1a, blue colors). *M. thermoautotrophicum* and other methanogens are described to encode methylcobalamin:homocysteine methyltransferase (core-MetE_Archaea_)^33^, a protein of 308 amino acids that is also homologous to the C-terminal part of tr-MetE proteins. *In vitro* experiments showed, that core-MetE_Archaea_ utilizes methylated corrinoids for the methylation of homocysteine, similar to what we now found for the core-MetE_CBDB_. Schröder and Thauer concluded that soluble methylcobalamin is unlikely the physiological methyl group donor and hypothesized that a corrinoid protein with yet unknown function could play this role. The gene products of *MTH124* or *MTH1156* were proposed as possible candidates*. MTH1156* encodes MtrH, a protein with sequence similarity to the 5-methyl-THF-Glu-binding domain of MetH (26% identity)^33^. MtrH is part of the methyl-tetrahydromethanopterin-coenzyme M methyltransferase complex and catalyzes the methylation of cob(I)alamin to methylcob(III)alamin using methyl-tetrahydromethanopterin as methyl group donor^62^. In contrast to methyl-THF biosynthesis in *Dehalococcoides* strains, methanopterin biosynthesis, a functional equivalent to THF in archaea, is fully encoded in all methanogenic archaea^49,63^. The methyl group of methionine in methanogenic archaea is derived from methyl-tetrahydromethanopterin^49,64^, which might be the primary methyl donor of archaeal methylcobalamin:homocysteine methyltransferases.

In conclusion, our findings show that bacterial core-MetE_CBDB_ homologs together with archaeal core-MetE representatives form a basal group of methionine synthases using methylcobalamin *in vitro* as co-substrate. Due to the fact that organisms encoding core-MetE enzymes are slowly growing strict anaerobes with strongly conserved ancient traits, we speculate that the core-MetE homologs are similar to an ancient methionine synthase encoded already in the genome of a predecessor of LUCA and therefore basal to both archaea and bacteria. We speculate that such basal methionine synthases were active in the metabolism of ancient microorganisms using methylcobalamin-containing proteins as methyl donor. Core-MetE_CBDB_ is the first biochemically described bacterial representative of these core-MetE proteins resembling the methylcobalamin:homocysteine methyltransferase from *M. thermoautotrophicum*^33^. Tr-MetE proteins appear to have evolved by duplications of core-MetE and subsequently acquired the capacity to bind folate at the N-terminal part. In our phylogenetic analysis tr-MetE clusters with archaeal core-MetE genes (Fig. 1a).

## Materials and Methods

### General

All chemicals were purchased from Sigma-Aldrich (Munich, Germany) or Carl Roth (Karlsruhe, Germany). Whenever methionine or homocysteine are mentioned in the text, the L-form is meant. Chemicals used for mass spectrometry were obtained in LC-MS grade from Carl Roth. Pteroyltri-γ-L-glutamic acid (PteGlu_3_) was acquired from Schircks Laboratories (Jona, Switzerland). Restriction enzymes, DNA polymerase, DNA and protein standards were obtained from New England BioLabs (Frankfurt/Main, Germany). Oligonucleotides and sequencing services were provided by Seqlab (Göttingen, Germany). All oligonucleotide primers, plasmids and strains used in this study are listed in Supplementary Tables 1 and 2. Anaerobic experiments were performed in a COY glovebox (Grass Lake, USA).

### Bioinformatics

The structural model of CbdbA481 (core-MetE_CBDB_) was calculated using the I-TASSER server^65^. Broadly defined, the server aligns the template protein with proteins of similar folds or with super-secondary structures from the PDB library by LOMETS. The overlay of core-MetE_CBDB_ and tr-MetE from *N. crassa* was generated with PyMOL^66^. The amino acid sequences of tr-MetEs were trimmed approximately at the position 370. For the multiple sequence alignment and construction of the phylogenetic tree, only the C-termini of truncated tr-MetEs from bacteria, yeast and complete amino acid sequences of core-MetEs from archaea, *Chloroflexi* and *Clostridiales* were used. MEGA7^67^ was used to calculate multiple amino acid sequence alignments using the implemented MUSCLE algorithm with default settings^68^. The evolutionary relationship between different methionine synthase amino acid sequences was inferred by using the Maximum Likelihood method based on the JTT matrix model^69^. Evolutionary distances were computed using Poisson correction and are expressed as the number of amino acid substitutions per site^70^.

### Construction of expression and complementation plasmids

Based on pBAD30, expression and complementation plasmids were generated as described in supplementary information. The resulting plasmids pBAD_MetE and pBAD_CbdbA481 were used for the complementation experiments as well as for the heterologous production and purification of tandem-repeat MetE (tr-MetE_Eco_) from *E. coli* and core-MetE_CBDB_ from *D. mccartyi* strain CBDB1.

### Production and purification of recombinant tr-MetE_Eco_ and core-MetE_CBDB_

A preculture of *E. coli* DH10B containing pBAD30_MetE or pBAD30_CbdbA481 was set up in Luria-Bertani (LB) medium containing 100 µg mL^−1^ ampicillin and grown overnight at 37 °C and 140 rpm. On the following day, 1% (v/v) of the overnight culture was used to inoculate fresh LB medium containing the appropriate antibiotic. The cultures were grown at 37 °C under agitation at 140 rpm until the OD_600_ reached 0.4–0.5. Then, the production of either tr-MetE_Eco_ or core-MetE_CBDB_ was induced by the addition of 0.05% (w/v) L-arabinose. Additionally, the medium was supplemented with 1 mM ZnSO_4_. MetE and CbdbA481 were produced for 5 h at 37 °C and 140 rpm. Then, the cells were harvested by centrifugation and washed with 50 mM Tris/HCl, pH 7.5. Purification of tr-MetE_Eco_ and core-MetE_CBDB_ was performed under anoxic conditions in an anaerobic chamber. Both enzymes were purified by anion exchange chromatography using a MonoQ 5/50 GL column connected to an ÄKTA purifier FPLC system (GE Healthcare Life Sciences) as described in detail in the supplementary information.

### SDS-PAGE and native PAGE

The purity of core-MetE_CBDB_ and tr-MetE_Eco_ protein preparations was evaluated by 10% SDS-PAGE. In addition, the oligomeric state of both proteins was investigated *via* 10% discontinuous native PAGE^71^.

### Protein identification from SDS-PAGE by LC-MS/MS

Qualitative identification of the purified core-MetE_CBDB_ and tr-MetE_Eco_ proteins was conducted mass spectrometrically. Therefore, protein bands at the height of 38 kDa and 80 kDa were excised from 10% SDS-PAGE gels. Acetonitrile, 10 mM DTT and 100 mM iodoacetamide were used to destain, to reduce and to alkylate the proteins within the gel slices. Subsequently, the proteins were digested with 0.1 µg trypsin (Promega) at 37 °C for 18 h. The resulting peptides were extracted from the gel matrix with 50% (v/v) acetonitrile and 5% (v/v) formic acid and dried. The peptides were again dissolved in 10 µL 0.1% formic acid and subsequently desalted using C_18_ ZipTip Pipette Tips (Merck Millipore) and dried in a vacuum centrifuge. Prior analysis, the peptides were resuspended in 20 µL 0.1% formic acid. Samples were analyzed on an LC-MS/MS system composed of a nano-UPLC system (UltiMate 3000 RSLCnano System, Thermo Fisher Scientific) equipped with an Acclaim PepMap 100 75 µm × 25 cm C_18_ column and connected to an Orbitrap Fusion mass spectrometer (Thermo Fisher Scientific) *via* an electrospray ion source (TriVersa NanoMate, Advion). Sample volumes of 5 µL were injected onto the column and separated applying a flow rate of 0.3 µL min^−1^ with the aid of a 60 min gradient from 3.2% to 44% acetonitrile in water containing 0.1% formic acid. The mass spectrometer was operated in positive-ionization mode. The spray voltage was set at 2.2 kV and an electron spray ionization source temperature at 220 °C. Full MS1 scans were obtained over a mass range of 300–2000 m/z and the resolution in the Orbitrap was set to 240,000. The most intense ions (threshold ion count above 5.0 × 10^4^) were selected for fragmentation with the quadrupole, setting the isolation window to 1.6 m/z. Ions were fragmented by ETciD (ETD reaction time 100 ms, CID collision energy 35%). The resulting fragment ion spectra were obtained achieved in the Orbitrap at a resolution of 60,000 and a maximum injection time of 120 ms.

### Protein and peptide identification

The raw mass spectrometric data were converted to mgf-files using ProteoWizard MSConvert v3.0^72^. The software SearchGUI (v3.3.5)^73^ and the OMSSA search algorithm were used for peptide identification. Mass spectrometric data were searched against the *E. coli* proteome database obtained from UniProt (Taxon identifier 316385). A precursor ion mass tolerance of 10 ppm was used at the MS1 level and up to two missed cleavages were allowed. The fragment ion mass tolerance was set to 0.2 Da for the Orbitrap MS2 detection. The oxidation of methionine was considered as variable modification and carbamidomethylation on cysteines as fixed modification. The false discovery rate (FDR) in peptide identification was limited to a maximum of 0.01 by using a decoy database. The analyzed data were visualized with the PeptideShaker software (v1.16.27) (CompOmics, Ghent University)^74^.

### Photometric analysis of methylcob(III)alamin binding to core-MetE_CBDB_

The binding of methylcob(III)alamin to core-MetE was followed spectrophotometrically in the range between 250 and 800 nm (0.5 nm steps) in 50 mM Tris/HCl (pH 6.5), 150 mM NaCl and 10% glycerol. First, the UV/Vis spectrum of 10 µM free methylcob(III)alamin was recorded. To assess the binding mode of methylcob(III)alamin to core-MetE_CBDB_, the protein solution was mixed with methylcob(III)alamin in a 1:1 stoichiometry (10 µM each). The UV/Vis spectra of free and bound methylcob(III)alamin were compared.

### Enzyme activity assay with methylcob(III)alamin as methyl group donor

Enzyme activity assays were set up at dim light under strictly anoxic conditions. The standard enzyme assay mix contained 50 mM Tris/HCl (pH 6.5), 150 mM NaCl, 10% glycerol, 0.5 mM methylcob(III)alamin and 0.1 µM enzyme. After 5 min preincubation at room temperature, the reaction was started by the addition of 2 mM D,L-homocysteine. The reaction was photometrically monitored either at 524 nm indicating the consumption of methylcob(III)alamin (ε_524_ = 6,200 M^−1^ cm^−1^) or at 681 nm indicating the formation of cob(I)alamin (ε_681_ = 1,200 M^−1^ cm^−1^) (Fig. 2a and Supplementary Figure 2)^75^. Enzyme kinetics of core-MetE_CBDB_ were performed at concentrations of either 0.5 mM methylcob(III)alamin or 2 mM D,L-homocysteine while the concentration of the second substrate was varied.

### Synthesis of (6R,S)-5-methyl-5,6,7,8-tetrahydropteroyltri-γ-L-glutamic acid (5-methyl-THF-Glu_3_)

The synthesis of 5-methyl-THF-Glu_3_ was accomplished from commercially available PteGlu_3_ under anoxic conditions following the modified protocol of Yeo and Wagner^76^ and as described in detail in the supplementary information. 5-methyl-H_4_PteGlu_3_ was stored at −20 °C.

### Enzyme activity assay with 5-methyl-THF-Glu_3_ as methyl group donor

Enzyme assays were performed under strictly anoxic conditions. The standard assay was set up in 25 mM Tris/HCl (pH 7.2) or 50 mM KH_2_PO_4_/K_2_HPO_4_ (pH 7.2), 100 µM MgSO_4_, 100 µM ZnSO_4,_ 10 mM dithiothreitol (DTT), 2 mM D,L-homocysteine and 150 µM 5-methyl-THF-Glu_3_. The reaction was started by the addition of 0.25 µM tr-MetE_Eco_ or core-MetE_CBDB_. After an incubation time of 60 min at 37 °C, the reactions were stopped by heat denaturation at 80 °C for 10 min, then centrifuged at 15,000 rpm for 5 min (Eppendorf Centrifuge 5424 R) and analyzed by HPLC. A negative control under same conditions without protein was run to evaluate abiotic transformation of 5-methyl-THF-Glu_3_. 5-methyl-THF-Glu_3_, other folate derivatives and PteGlu_3_ were analyzed with a JASCO HPLC 2000 series system equipped with an Equisil BDS C_18_ column (250 × 4.6 mm, 5 μm; Dr. Maisch HPLC GmbH, Ammerbuch-Entringen/Germany) following the modified protocol of Patring^77^. The identities of PteGlu_3_, 5-methyl-THF-Glu_3_ and L-methionine were confirmed *via* liquid chromatography-mass spectrometry in direct injection mode.

### Generation of *E. coli* knockout strain

The *E. coli* DH5α (Δ*metE::kan)* knockout strain was generated using the Quick & Easy *E. coli* Gene Deletion Kit (GeneBridges GmbH, Heidelberg, Germany) according to the manufacturer’s protocol^78^. Hereby, *metE* gene in *E. coli* DH5α was replaced by a linear kanamycin cassette. The introduction of the kanamycin cassette allowed to screen for the knockout strain on 20 µg mL^−1^ kanamycin agar plates.

### *In vivo* complementation of *E. coli* DH5α (*ΔmetE::kan*) and cultivation procedure

The *metE-*deficient *E. coli* DH5α (*E. coli* DH5α (*ΔmetE::kan*)) strain was transformed with pBAD30 (negative control), pBAD30_MetE (positive control) or pBAD30_CbdbA481. The first subculture was grown in 5 mL LB medium with 100 µg mL^−1^ ampicillin and 20 µg mL^−1^ kanamycin at 37 °C and 140 rpm overnight. An inoculum of 1% (v/v) of the first subculture was then used to inoculate the second subculture of 5 mL M9 minimal medium^79^ supplemented with 1 mM MgSO_4_, 0.1 mM CaCl_2_, 10 µM FeCl_3_/EDTA, 1.2 mM thiamine, 0.3 mM L-leucine, 0.4% (v/v) glycerol and 0.4 µM cyanocobalamin, that was grown at 37 °C and 140 rpm overnight. Then, several 10 mL-tubes of fresh M9 medium containing all supplements except cyanocobalamin were inoculated with 1% (v/v) of the second subculture. The following main cultures were set up:

- *E. coli* DH5α wild type with or without 0.4 µM cyanocobalamin,
- *E. coli* DH5α (*ΔmetE::kan*) with or without 0.4 µM cyanocobalamin,
- *E. coli* DH5α (*ΔmetE::kan*) + pBAD30_MetE with or without 0.4 µM cyanocobalamin and with or without 0.05% (w/v) L-arabinose,
- *E. coli* DH5α (*ΔmetE::kan*) + pBAD_CbdbA481 with or without 0.4 µM cyanocobalamin and with or without 0.05% (w/v) L-arabinose.

The main cultures were then incubated at 37 °C and 140 rpm and growth was monitored by measuring the OD_600_. *E. coli* DH5α still encodes the arabinose operon. However, arabinose at a concentration of 0.05% (w/v) sufficed to induce the production of core-MetE_CBDB_ and tr-MetE_Eco_. In our experiments, reciprocal metabolism leads to preferential use of glycerol instead of arabinose.

## Supplementary information

Supplementary information.
