# Supplementary material for: Identification and characterization of a bacterial core methionine synthase
Source: Sci Rep. 2020 Feb 7;10:2100. doi: 10.1038/s41598-020-58873-z (PMC7005905; doi:10.1038/s41598-020-58873-z)
Supplement: Supplementary file 1 — Supplementary information. [file 41598_2020_58873_MOESM1_ESM.docx]

# Supplementary Material for the manuscript

# Identification and characterization of a bacterial core methionine synthase

Darja Deobald^1,2^, Rafael Hanna^1,3^, Shahab Shahryari^2^, Gunhild Layer^1,3^, Lorenz Adrian^2,4*^

^1^Leipzig University, Institute of Biochemistry, Brüderstraße 34, 04103 Leipzig, Germany

^2^Helmholtz Centre for Environmental Research – UFZ, Isotope Biogeochemistry, Permoserstraße 15, 04318 Leipzig, Germany

^3^Freiburg University, Institute of Pharmaceutical Sciences, Stefan-Meier-Straße 19, 79104 Freiburg im Breisgau, Germany

^4^Technische Universität Berlin, Chair of Geobiotechnology, Ackerstraße 76, 13355 Berlin, Germany

^*^To whom correspondence should be addressed:

Lorenz Adrian, Helmholtz Centre for Environmental Research - UFZ, Isotope Biogeochemistry, Permoserstraße 15, 04318 Leipzig, Germany, Phone +49 (0) 341 235 1435, Fax +49 (0) 341 235 1443, lorenz.adrian@ufz.de

## Materials and Methods

**Construction of pBAD30_MetE and pBAD30_CbdbA481 plasmids.** Expression and complementation plasmids pBAD30_MetE and pBAD30_CbdbA481 were generated based on pBAD30 vector. EcoRI and HindIII restriction sites in pBAD30 were selected for cloning the *metE* gene from *E. coli* or the cbdbA481 sequence from *D. mccartyi* strain CBDB1 into the vector. Both genes were PCR-amplified using Q5^®^ High-Fidelity DNA polymerase and oligonucleotide primers A481_fw and A481_rev or metE_fw and metE_rev containing 15 nucleotide overhangs (Table S1). Besides the homologous overhangs, A481_fw and metE_fw primers contained a ribosome binding site that is absent in the original pBAD30 ^1^. The obtained PCR fragments were purified using the GeneJET PCR Purification Kit (Thermo Fisher Scientific) and were cloned into linearized pBAD30 using the In-Fusion^®^ HD Cloning Kit (Takara Bio USA, Inc.) according to the manufacturer’s instructions.

**Purification of the tandem-repeat type MetE from *E. coli* (tr-MetE_Eco_) and core-MetE_CBDB_ from *D. mccartyi* strain CBDB1**. All purification steps were performed under anoxic conditions. Frozen cell pellets were transferred into an anaerobic chamber. Here, the cell pellet with produced core-MetE_CBDB_ was resuspended in 50 mM Tris/HCl, pH 7.5 and the cell pellet with produced tr-MetE_Eco_ in 100 mM KH_2_PO_4_/K_2_HPO_4_, pH 7.2. Subsequently, the cells were disrupted by a FastPrep-24^TM^ 5G (MP Biomedicals, Santa Ana, CA, USA) at 6 m s^-1^ for 2 x 30 s using 200 µm silica beads. Soluble protein fractions were obtained by centrifuging the crude extracts at 4 °C and 40,000 rpm for 1 h (Beckman Coulter Optima L-90K). Both, tr-MetE_Eco_ and core-MetE_CBDB_ were purified by anion exchange chromatography using a MonoQ 5/50 GL column connected to an ÄKTA purifier FPLC system (GE Healthcare Life Sciences). For that, both soluble protein fractions were applied to the column equilibrated either with 50 mM Tris/HCl, pH 7.5 for core-MetE_CBDB_ or with 100 mM KH_2_PO_4_/K_2_HPO_4_, pH 7.2 for tr-MetE_Eco_. After protein binding, the column was washed with the corresponding buffer (50 mM Tris/HCl for core-MetE_CBDB_ and with 100 mM KH_2_PO_4_/K_2_HPO_4_ for tr-MetE_Eco_). Core-MetE_CBDB_ was eluted using a linear NaCl gradient (0 to 400 mM NaCl within 40 mL) at a flow rate of 1 mL min^−1^. Elution of tr-MetE_Eco_ was achieved by applying a linear KH_2_PO_4_/K_2_HPO_4_ gradient (0.1 to 1 M within 50 mL). Elution of the proteins was followed by monitoring the absorbance at 280 nm. As determined by SDS-PAGE, core-MetE_Dhc_ eluted at approximately 60 mM NaCl. tr-MetE_Eco_ eluted at approximately 200 mM KH_2_PO_4_/K_2_HPO_4_. Fractions containing the target protein were pooled and concentrated with Amicon^®^ Ultra-4 centrifugal filter units with a corresponding molecular weight cutoff (30 K for core-MetE_CBDB_ and 60 K for tr-MetE_Eco_). Protein concentrations were determined by the Bradford assay ^3^.

**Synthesis of 5-methyl-THF-Glu_3_.** 5-methyl-THF-Glu_3_ was synthesized from PteGlu_3_ under anoxic conditions following a protocol modified from Yeo and Wagner ^8^. PteGlu_3_ was dissolved in 100 mM NaOH to a final concentration of 10 mM and 200 µL of this 10 mM PteGlu_3_ solution were mixed with 4 mL of 6 mM Tris/HCl, pH 7.8. Next, PteGlu_3_ was reduced to H_4_PteGlu_3_ with 400 µL of 5.28 M NaBH_4_ at 45 °C for 30 min. After this, the pH was adjusted to 5.0 with 5 M formic acid to eliminate excess NaBH_4_. Subsequently, the pH was titrated back to 7.5 with 1 M NaOH. Afterwards, 800 µL of 0.06% formaldehyde were added and the reaction mixture was incubated at 45 °C for 15 min to form 5,10-methylene-THF-Glu_3_. The final reduction step with 800 µL of 5.28 M NaBH_4_ was conducted in the dark at 45 °C for 1 h and resulted in the formation of 5‑methyl-THF-Glu_3_. The reaction mixture was cooled to room temperature and 60 µL of 14 M β‑mercaptoethanol was added. Then, the pH was again adjusted to 5.0 with 5 M formic acid and to pH 7.5 with 1 M NaOH. The synthesis of 5‑methyl-THF-Glu_3_ was verified photometrically by recording the UV/Vis absorption spectrum, and its concentration was determined by measuring the absorbance at 290 nm (ε_290_= 31,700 M^-1^ cm^-1^) ^9^. Furthermore, the identity of the educt PteGlu_3_ and the product 5‑methyl-THF-Glu_3_ was confirmed *via* HPLC and LC-MS (Supplementary Figure 5). The 5‑methyl-THF-Glu_3_ preparation was stored at -20 °C.

**HPLC analysis of 5-methyl-THF-Glu_3_ and derivatives.** 5-methyl-THF-Glu_3_, other folic acid derivatives and PteGlu_3_ were analyzed with a JASCO HPLC 2000 series system equipped with an Equisil BDS C_18_ column (250 x 4.6 mm, 5 μm; Dr. Maisch HPLC GmbH, Ammerbuch-Entringen, Germany). For the detection of folates and PteGlu_3_ a fluorescence detector (excitation: 290 nm, emission: 360 nm) and a diode array detector (absorbance: 290 nm) were used. The folates were analyzed at a flow rate of 0.5 mL min^-1^ following a protocol modified from Patring *et al.* ^10^. The column was equilibrated with solvent A consisting of 0.1% (v/v) aqueous trifluoroacetic acid prior to analysis. Elution of compounds was achieved with solvent B consisting of acetonitrile acidified with 0.1% (v/v) trifluoroacetic acid. After injecting 50 µL of each sample, a linear gradient was run reaching 30% of solvent B after 25 min. Afterwards, the content of solvent B was increased to 100% within 5 min. The column was washed with 100% solvent B for further 5 min. Subsequently, the initial conditions were re-established within 1 min. Finally, the column was re-equilibrated with solvent A for 10 min.

**LC-MS analysis of 5-methyl-THF-Glu_3_ and derivatives.** The identities of PteGlu_3_ ([M+H]^+^= 700.2332 m/z), 5-methyl-THF-Glu_3_ ([M+H]^+^= 718.7571 m/z) and methionine ([M+H]^+^= 150.0583 m/z) were confirmed *via* LC-MS in direct injection mode using an Orbitrap Fusion mass spectrometer (Thermo Scientific). The mass spectrometer was run in positive-ionization mode with the spray voltage set at 2.2 kV and source temperature at 220 °C. For full scan MS1 mode, the instrument operated over a mass range of 50-2000 m/z with detection at a resolution of 120,000. Subsequently, the MS1 raw data were converted to mzML-files using ProteoWizard MSConvert v3.0 ^5^ and further analyzed with OpenMS TOPPView software ^11^.

**References**

1. Guzman, L. M., Belin, D., Carson, M. J. & Beckwith, J. Tight regulation, modulation, and high-level expression by vectors containing the arabinose pBAD promoter. *J Bacteriol* **177,** 4121–4130; 10.1128/jb.177.14.4121-4130.1995 (1995).

2. Harwood, C. R. & Cutting, S. M. in *Molecular biological methods in Bacillus*, edited by C. R. Harwood & S. M. Cutting (Wiley, Chichester, 1990), Vol. 1, p. 548.

3. Bradford, M. M. A rapid and sensitive method for the quantitation of microgram quantities of protein utilizing the principle of protein-dye binding. *Anal Biochem* **72,** 248–254; 10.1016/0003-2697(76)90527-3 (1976).

4. Ornstein, L. & Davis, B. J. Disc electrophoresis-I: background and theory. *Ann NY Acad Sci* **121,** 321–349 (1964).

5. Holman, J. D., Tabb, D. L. & Mallick, P. Employing ProteoWizard to convert raw mass spectrometry data. *Curr Protoc Bioinformatics* **46,** 13.24.1-9; 10.1002/0471250953.bi1324s46 (2014).

6. Vaudel, M., Barsnes, H., Berven, F. S., Sickmann, A. & Martens, L. SearchGUI: an open-source graphical user interface for simultaneous OMSSA and X!Tandem searches. *Proteomics* **11,** 996–999; 10.1002/pmic.201000595 (2011).

7. Vaudel, M. *et al.* PeptideShaker enables reanalysis of MS-derived proteomics data sets. *Nature Biotechnol* **33,** 22-24; 10.1038/nbt.3109 (2015).

8. Yeo, E. J. & Wagner, C. Purification and properties of pancreatic glycine N-methyltransferase. *J Biol Chem* **267,** 24669–24674 (1992).

9. Gupta, V. S. & Huennekens, F. M. Preparation and properties of crystalline 5-methyltetrahydrofolate and related compounds. *Arch Biochem Biophys* **120,** 712–718 (1967).

10. Patring, J. D. M., Jastrebova, J. A., Hjortmo, S. B., Andlid, T. A. & Jägerstad, I. M. Development of a simplified method for the determination of folates in baker's yeast by HPLC with ultraviolet and fluorescence detection. *J Agric Food Chem* **53,** 2406–2411; 10.1021/jf048083g (2005).

11. Sturm, M. & Kohlbacher, O. TOPPView: an open-source viewer for mass spectrometry data. *Journal of proteome research* **8,** 3760–3763; 10.1021/pr900171m (2009).

12. Off, M. K. *et al.* Ultraviolet photodegradation of folic acid. *J Photochem Photobiol B, Biol* **80,** 47–55 (2005).

13. Steindal, A. H., Juzeniene, A., Johnsson, A. & Moan, J. Photodegradation of 5-methyltetrahydrofolate: biophysical aspects. *Photochem Photobiol* **82,** 1651–1655 (2006).

14. Donaldson, K. O. & Keresztesy, J. C. Naturally occuring forms of folic acid. *J Biol Chem* **237,** 3815–3819 (1962).

15. Chen, J., Li, Y., Zhang, K. & Wang, H. Whole-genome sequence of phage-resistant strain *Escherichia coli* DH5α. *Genome Announc* **6,** e00097-18; 10.1128/genomeA.00097-18 (2018).

## Supplementary Figures

**
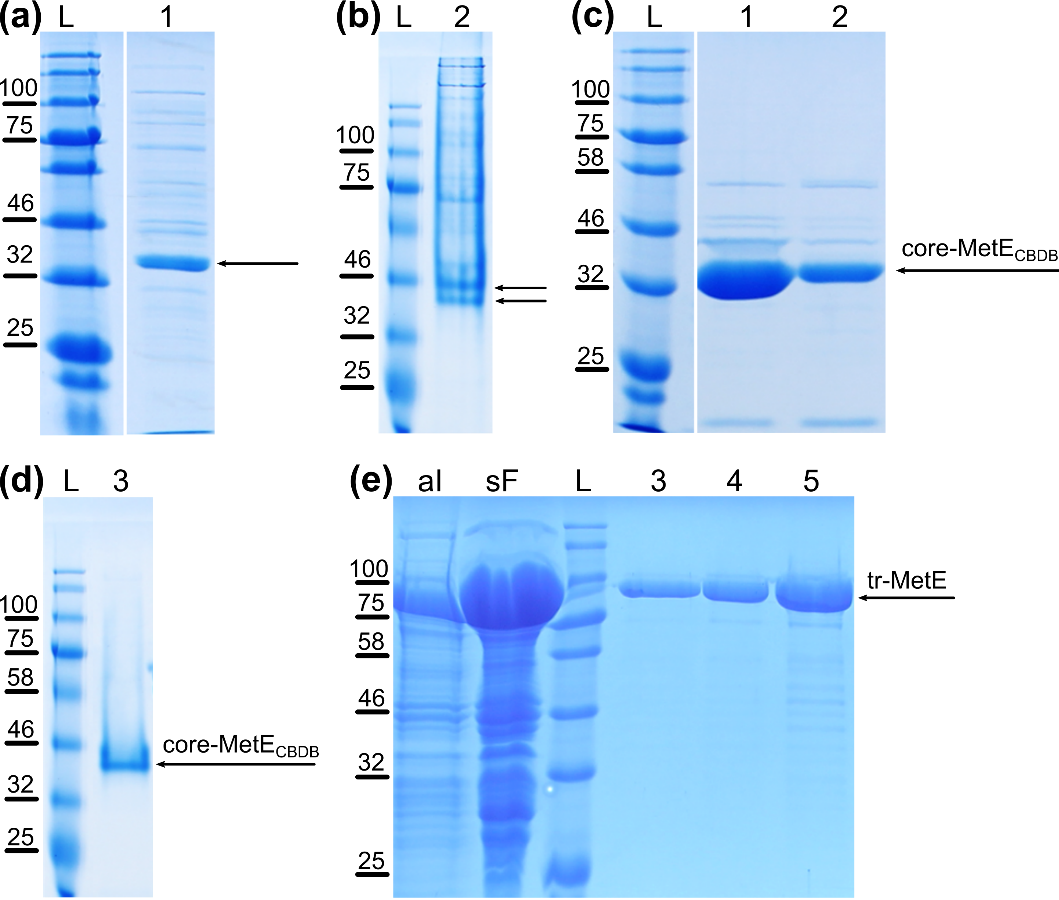
**

**Supplementary Figure 1.** **Purification and analysis of the cbdbA481 gene product from *Dehalococcoides mccartyi* strain CBDB1. (a)** SDS-PAGE analysis of streptavidin-tagged core-MetE_CBDB_ after affinity purification using a Strep-Tactin gravity column (IBA GmbH). 1: core-MetE_CBDB_-containing elution fraction, **(b)** discontinuous native PAGE analysis of streptavidin-tagged core-MetE_CBDB_ after affinity purification. 2: the presence of two bands for streptavidin-tagged core-MetE_CBDB_ (two arrows) indicated the occurrence of two different tertiary structures. **(c)** SDS-PAGE analysis of untagged core-MetE_CBDB_ purified by anion exchange chromatography using a MonoQ 5/50 GL column (GE Healthcare Life Sciences). 1-2: Core-MetE_Dhc_-containing elution fractions. **(d)** Analysis of the oligomerisation state of untagged core-MetE_CBDB_ by discontinuous native PAGE. Core-MetE_CBDB_ is marked with an arrow. **(e)** SDS-PAGE analysis of untagged tr-MetE_Eco_ purified by anion exchange chromatography using a MonoQ 5/50 GL column (GE Healthcare Life Sciences). aI: proteins after induction, sF: soluble protein fraction, 3-5: elution fractions and L: Prestained Protein Ladder, Broad Range (11-190 kDa, New England BioLabs).

**
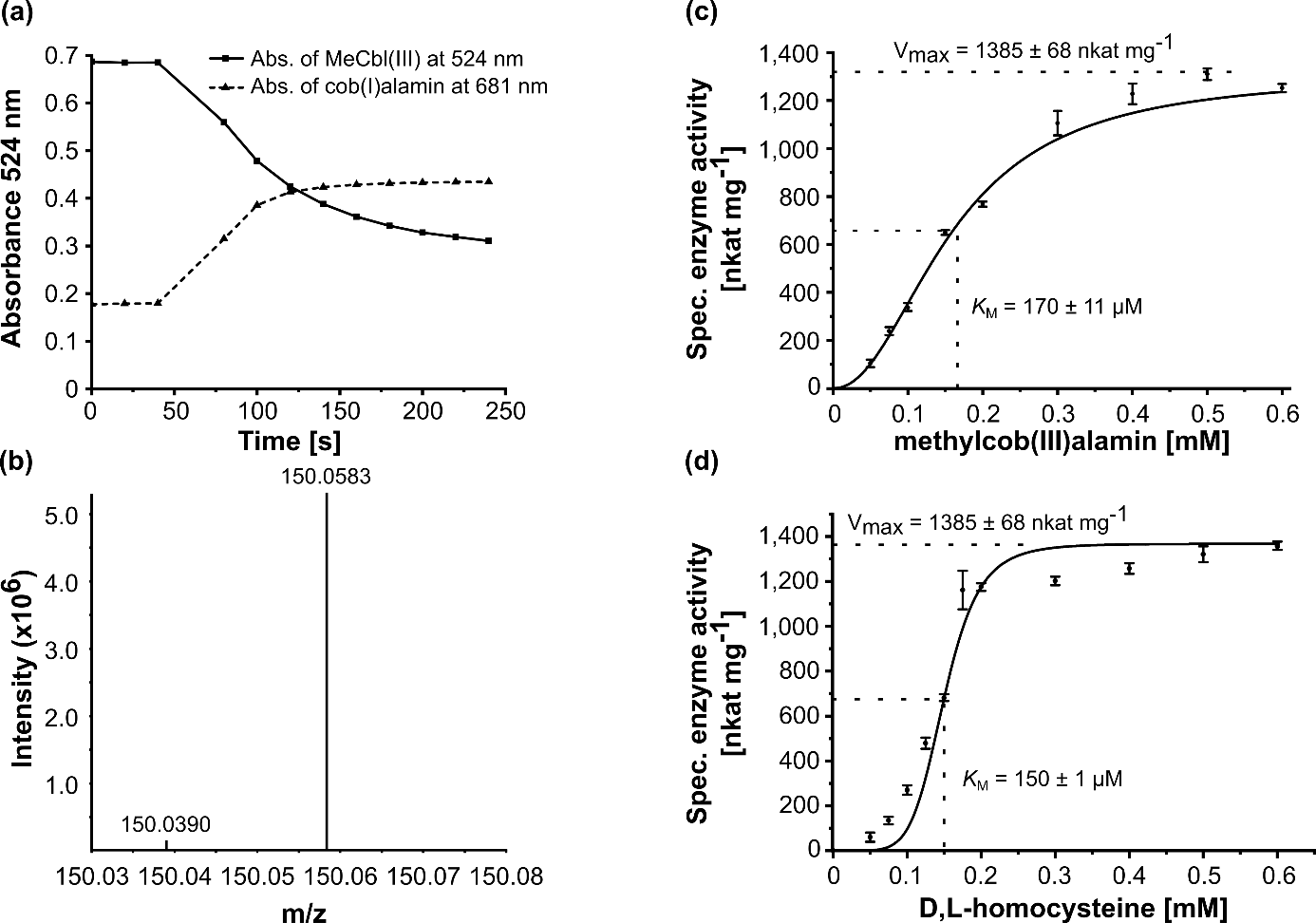
**

**Supplementary Figure 2. Enzyme activity of methionine synthase core-MetE_CBDB_ from *D. mccartyi* strain CBDB1. (a)** The enzymatic reaction was followed in the presence of 0.1 µM core-MetE_CBDB_ and 0.15 mM methylcob(III)alamin by measuring the absorbance changes at 524 nm (solid line) and 681 nm (dashed line) representing the consumption of methylcob(III)alamin and the formation of cob(I)alamin, respectively. The reaction was started at t = 40 s by the addition of 2 mM D,L-homocysteine. **(b)** Formation of methionine ([M+H]^+^ = 150.0583 m/z) was confirmed *via* LC-MS. **(c)** Dependence of core-MetE_CBDB_ methyltransferase activity on the methylcob(III)alamin concentration. The specific enzyme activity was followed by the change of absorbance of methylcob(III)alamin at 524 nm. *V*_max_ = 1385 ± 68 nkat mg^-1^ and *K*_M_ = 170 ± 11 µM were calculated according to a Hill-Fit plot with R^2^ = 0.997. **(d)** Rate of methyl group transfer from methylcob(III)alamin to homocysteine depending on the D,L-homocysteine concentration. The specific enzyme activity was followed by the change of absorbance at 524 nm. *V*_max_ = 1356 ± 42 nkat mg^-1^ and *K*_M_ = 150 ± 1 µM were calculated according to a Hill-Fit plot with R^2^ = 0.999.


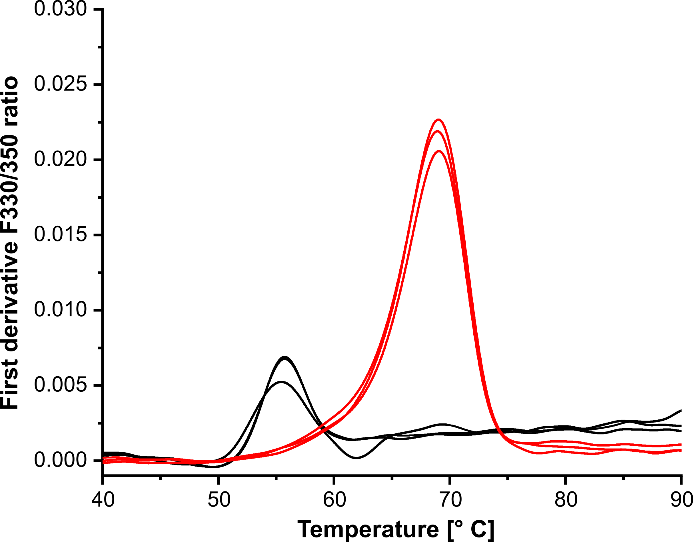


**Supplementary Figure 3. Determination of the melting temperatures (T_m_) of purified core-MetE_CBDB_ (red) from *D. mccartyi* strain CBDB1 and tr-MetE_Eco_ from *E. coli* (black) using the nanoDSF principle.** T_m_ was calculated from the first derivative of the F_330_/F_350_ ratio of the intrinsic tryptophan fluorescence. T_m_(core-MetE_CBDB_) = 68.8±0.0 °C, T_m_(tr-MetE_Eco_) = 55.8±0.2 °C. Shown are the curves of three independent measurements for both enzymes. The T_m_ values are given as means with standard deviation.

##
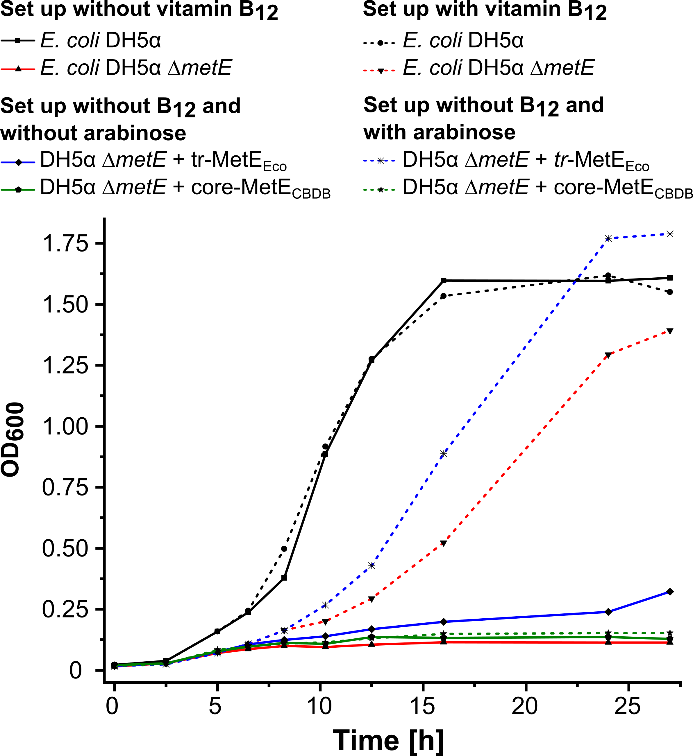


**Supplementary Figure 4.** **Growth behavior of *E. coli* DH5α wild type (wt) and ∆*metE* knockout (∆*metE*) strains and the ∆*metE* strain complemented with either tr-MetE_Eco_ or core-MetE_CBDB_ in the presence/absence of cyanocobalamin (vitamin B_12_) and the presence/absence of 0.05 % (w/v) L-arabinose.** The *metE*-deficient *E. coli* strain was not able to grow in cobalamin-free M9 medium (red solid line), but grew in the presence of vitamin B_12_ (red dotted line). The growth phenotype of the ∆*metE* strain was rescued by expressing tr-MetE_Eco_ using plasmid pBAD_MetE and arabinose induction (blue dotted line). In contrast, expression of core-MetE_CBDB_ from the pBAD_CbdbA481 vector did not complement the mutant strain under identical conditions (green dotted line). The growth experiments were qualitatively reproduced at least two times, shown are mean values of three independent cultures.


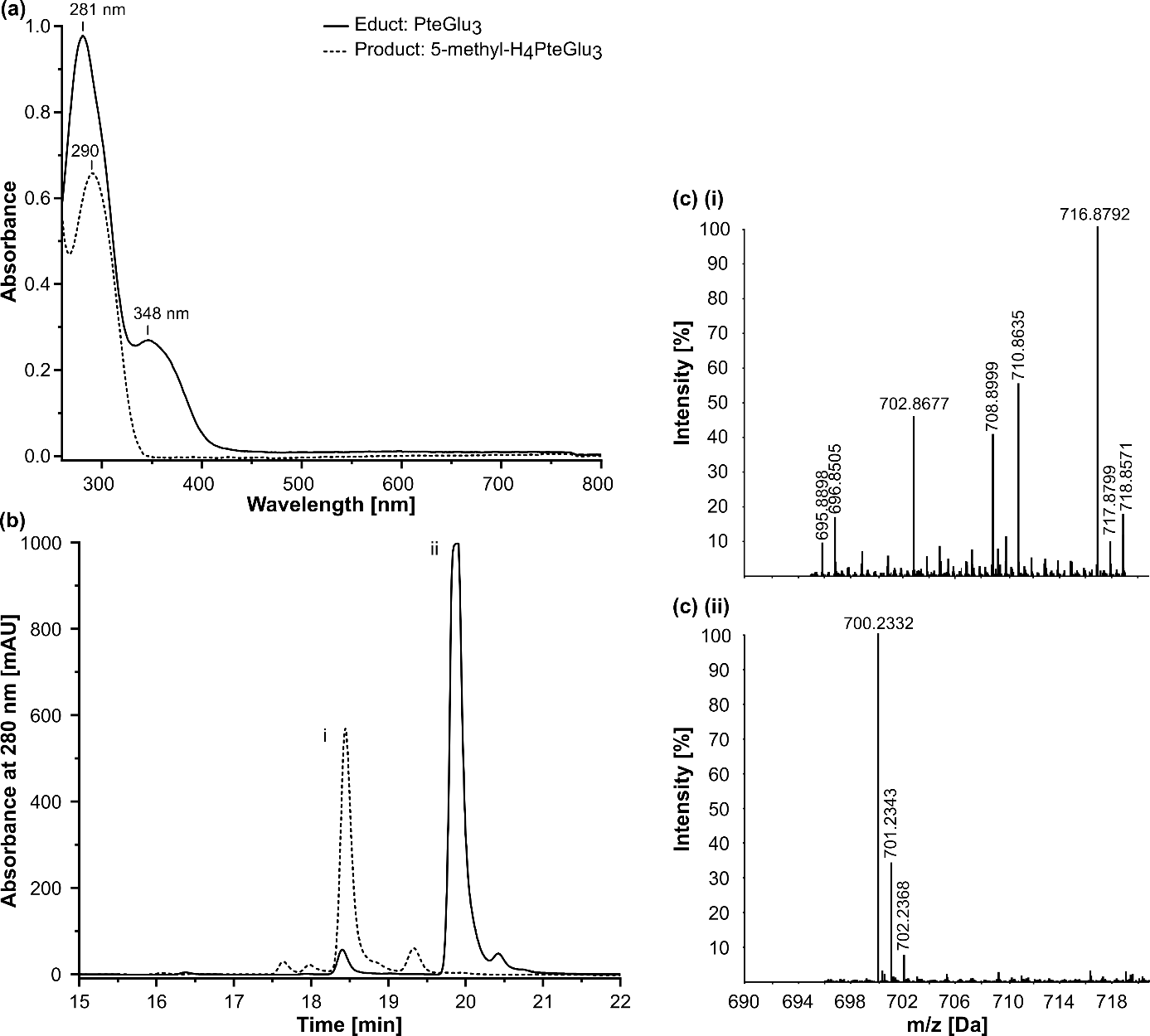


**Supplementary Figure 5. Synthesis of 5-methyl-H_4_PteGlu_3_ from PteGlu_3_.** **(a)** UV/Vis absorption spectra of PteGlu_3_ (solid line) with characteristic absorption maxima at 281 and 348 nm ^12^ and 5-methyl-THF-Glu_3_ (dashed line) exhibiting a single absorption maximum at 290 nm ^13^. **(b)** HPLC analysis of PteGlu_3_ (solid line) and 5-methyl-THF-Glu_3_ (dashed line). Elution of the compounds was followed by recording the absorbance at 280 nm. **(c)** The identity of the compounds was verified by LC-MS: **i)** 5-methyl-THF-Glu_3_ at [M+H]^+^ = 718.8571 m/z. 5-methyl-THF-Glu_3_ reacts in the presence of oxygen to 5-methyl-DHF-Glu_3_ ([M+H]^+^ = 716.8792 m/z) ^14^ and **ii)** PteGlu_3_ at [M+H]^+^ = 700.2332 m/z.

## Supplementary Tables

**Supplementary Table 1. Oligonucleotide primers used in this study.** 15 nt overhangs complementary to the EcoRI and HindIII digested pBAD30 are underlined. Introduced ribosome binding site is printed in italics. The start codon is highlighted in bold.

| Primers | Sequence | Application |
| --- | --- | --- |
| metE_fw | TTGGGCTAGCGAATT*AGGAGG*AATTAACC**ATG**ACAATATTGAATCAC | PCR amplification |
| metE_rev | CAAAACAGCCAAGCTTTACCCCCGACGCAAGTTCT | PCR amplification |
| A481_fw | TTGGGCTAGCGAATT*AGGAGG*AATTAACC**ATG**ACTAAGACCGATTTTTA | PCR amplification |
| A481_rev | CAAAACAGCCAAGCTTCATACTGTTCCGTGTTTCC | PCR amplification |

**Supplementary Table 2. Strains used in this study.**

| Strains | Genotype | Reference |
| --- | --- | --- |
| E. coli DH5α | *F^–^ ompT gal dcm lon hsdS_B_(r_B_^–^m_B_^–^) λ(DE3 [lacI lacUV5-T7p07 ind1 sam7 nin5]) [malB^+^]_K-12_(λ^S^)* | ^15^ |
| E. coli DH5α (ΔmetE::kan) | *F^–^ endA1 glnV44 thi-1 recA1 relA1 gyrA96 deoR nupG purB20 φ80dlacZΔM15 Δ(lacZYA-argF)U169, hsdR17(r_K_^–^m_K_^+^), λ^–^ ΔmetE::kan* | this study |
